# Risk-sharing agreements, present and future

**DOI:** 10.3332/ecancer.2018.823

**Published:** 2018-04-10

**Authors:** Francisco R Gonçalves, Susana Santos, Catarina Silva, Gabriela Sousa

**Affiliations:** 1 Portuguese Oncology Institute of Porto, 4200-072 Porto, Portugal; 2 Roche Farmacêutica Química Lda, 2720-410 Amadora, Portugal; 3 Eurotrials—Scientific Consultants, 1070-274 Lisbon, Portugal; 4 CISP, National School of Public Health, 1600-560 Lisbon, Portugal; 5 Portuguese Oncology Institute of Coimbra, 3000-075 Coimbra, Portugal

**Keywords:** risk sharing, agreement, price per combination, price per indication, access

## Abstract

Risk-sharing agreements between pharmaceutical companies and payers stand out as a recent practice, the use of which has been increasing in the case of innovative medicines, particularly in the field of oncology, which aims to ensure better budgetary control and a lower risk of spending on medicinal products without full evidence of clinical benefit.

In this article, the authors discuss the types of existing agreements, as well as those used in Portugal, their advantages, disadvantages and future challenges of implementation, as well as their potential role in access to therapeutic innovation, namely medicines for cancer treatment. For this purpose, a nonsystematic review of indexed and nonconventional literature was carried out.

There is a tendency for the risk-sharing agreements established between payers and pharmaceutical companies to include a component of monitoring the use of medicines and outcomes measurement, involving real life data collection. Portugal is no exception and, although most agreements are still financial in nature, there is already a strong desire for other agreements, in particular clinical outcomes based.

It is concluded that there is not yet a gold standard methodology in relation to the type of agreements to be practiced. Moreover, its opportunity cost, including the cost of implementation, remains to be scrutinised. However, regardless of the type of agreement, the advantages of adopting these agreements are well known, inevitably related with challenges of implementation. The need for an infrastructure to support information sharing is undisputed and urgent.

The future of therapeutic innovation and increased pressure on health budgets will require alternative, more flexible models, personalized reimbursement models that allow alignment of medicines prices with the value they deliver in treating the several diseases.

## Introduction

The need to curb the growth of health expenditures, particularly in the field of oncology, has been reflected in measures to restrain spending by policy makers, particularly in countries where the public sector has a predominant role in the provision/reimbursement of medicines. Several mechanisms have been used, such as the introduction of co-payments for pharmaceutical dispensing, control of profit margins with medical and pharmaceutical products, price referral and performance of health technology assessments (HTAs). At the same time, policymakers have been trying to find new ways to set the pricing and reimbursement of innovative medicines [1, 2]. One of the instruments, whose use has been growing, is the risk-sharing agreements (RSAs) [2, 3] between pharmaceutical companies and payers, aimed at guaranteeing access to innovation, but promoting the sustainability of the National Health Service (NHS). More specifically, these agreements allow patients access to innovative medicines in a context of uncertainty about their clinical benefit and cost effectiveness due to still limited and/or immature evidence, identifying patient groups where the drug is most effective and reducing the risk of unnecessary expenses by the payers when they are reimbursed [1, 2, 4, 5]. RSAs allow (a) mitigation of uncertainty about clinical outcomes and cost effectiveness of medicines by allowing conditional reimbursement of the drug, which is dependent on collecting additional evidence for final decision making, (b) basing reimbursement or payment on outcomes of the drug in the context of clinical practice, (c) restricting the use of the medicinal product to a sub-population of patients through selection or eligibility criteria where they are most effective and (d) managing the budgetary impact [4]. These agreements also allow the price of medicinal products to be aligned with the benefit they provide in a given therapeutic indication or in a particular combination of medicinal products, since different agreements may be implemented depending on the indication or association of medicinal products concerned.

Most of the RSAs have been used in the reimbursement of cancer medicines, since access to these medicines varies substantially between European countries (in particular with regard to the list of medicines that are accessible and the waiting time) and to the fact that their value for money (acceptable cost versus efficay/safety) is often perceived as ‘low’ (possibly, because the pivotal study and the first approval usually focus on the indication of the drug for advanced stages of the disease) [3].

The aim of this study is to characterise the existing types of RSAs, identifying their advantages and disadvantages, characterise the existing experience in Portugal and also to sensitise the scientific community to the need to define new pricing modalities based on the benefit the medicinal product delivers, namely in the field of oncology, and the indispensability of creating registries that capture the data required for the implementation of RSAs.

A nonsystematic literature review was carried out by one of the authors in January 2017, using search engines such as PubMed, using the keywords ‘risk sharing’, ‘managed entry agreements’, ‘indication value-based pricing’, ‘conditional reimbursement’, ‘multiple indication pricing’, ‘drug combination pricing’ and ‘patient access schemes’ for the period between 2010 and 2017. This review was also complemented by nonconventional literature (online reports, presentations at conferences, nonacademic/indexed journals) of the authors’ knowledge. The main focus was on the European context, but documentation has been reviewed with examples related to the United States, Australia and Canada. Only articles whose content was potentially relevant to the writing of this article were considered. A qualitative review of the articles was not carried out.

## Risk-sharing agreements

The concept of risk sharing is still relatively recent in the field of health policy; and therefore, remains a subject of dubious interpretation, both in terms of terminology and concept [1, 3, 5]. Several terms, definitions and taxonomies have appeared in the literature to classify these agreements [1–3, 5, 7, 8]. In this paper, we will follow the RSA, as defined by Health Technology Assessement International (HTAi) [3, 8] as ‘an agreement between the producer/manufacturer and the payer/provider that allows access (coverage/reimbursement) of a health technology under certain conditions. These agreements may use a variety of mechanisms to address uncertainty about technology performance or to manage technology adoption in order to maximise their effective use or to limit their budgetary impact’.

**Figure 1.**
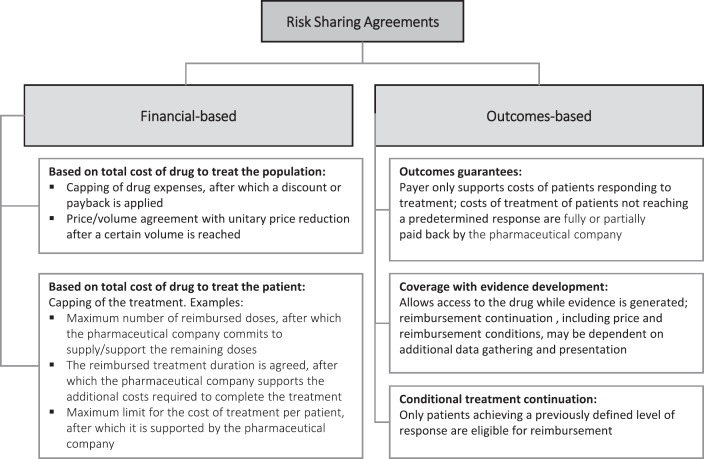
Taxonomy for risk-sharing agreements.

RSAs are divided into (a) financial agreements, where cost containment is defined merely on the basis of the price of the medicinal product or the cost of the treatment and (b) agreements based on clinical results, i.e., associated with the performance of the medicinal product in real clinical practice. In this type of RSA, there is an agreement between the payer/provider and the pharmaceutical company for the collection of real-world data and the payment is based on the observed results. Such agreements are most appropriate where there is uncertainty associated with the efficacy/safety of a given drug, allowing risk sharing between the payer and the pharmaceutical company. This type of RSA provides a great opportunity for the collection of data from real clinical practice (real-world data) [3, 9]. Figure 1 outlines the various types of existing agreements [2, 5, 7].

There are a number of reasons that justify and encourage the implementation of RSAs, namely improving the sustainability of the health system without impeding access to medicinal products for treatment in therapeutic areas with therapeutic gaps, such as oncology [3, 7]. However, like any other cost containment measure, there are also drawbacks. Table 1 summarises the advantages and disadvantages of RSAs [8–12].

It is noted that countries have adopted different types of agreements according to their purpose and the type of health system [1, 3, 5]. As in many cost containment measures, theory is more attractive than practice, with great variability of outcomes between and within the various health systems. In addition, the information on their objectives, methodologies and monitoring systems is still scarce, as is information on the real impact of RSAs, both socially and economically [3, 7]. However, according to a study carried out in 2010, one of the few that evaluates the benefit of RSAs, the introduction of these agreements seems to have contributed substantially to an improvement in the access of cancer medicines to Italian patients. In fact, it was verified that the median time for authorisation of oncological drugs with an RSA was 84 days, while for authorisations without risk sharing the median time was 343 days [1, 3, 9]. In addition, health professionals appear to be satisfied with the results of these agreements. A survey also conducted in Italy showed that one in two medical oncologists believes that RSAs are the ‘way forward’ [1].

**Figure 2.**
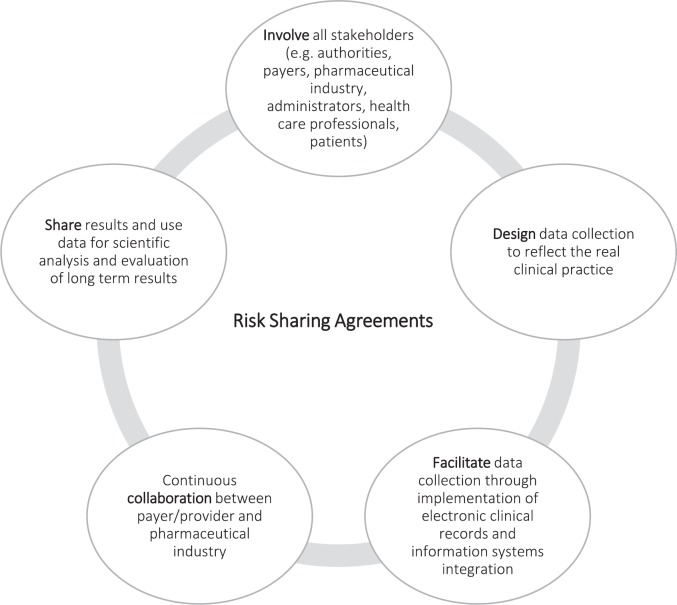
Recommendations for RSAs.

Although the implementation of RSAs in various countries is still relatively recent and information is limited, their experience could undoubtedly contribute to the success of future RSAs. It would be important to encourage the development of guidelines or best practices in the use of RSAs in light of those issued by the International Society for Pharmacoeconomics and Outcomes Research (ISPOR) [5] or by some working groups such as the Apollo Network in The Netherlands [4] or the Managed Entry Agreement Risk Analysis Framework [2] in the United Kingdom. Figure 2 summarises some of these recommendations [2, 4, 5, 11].

## Role of RSAs in medicines with multiple indications

It is common for the same health technology to provide clinical benefits in multiple therapeutic indications and in different sub-groups of patients, namely, cancer drugs approved for various types of cancer and in several lines of treatment. In fact, in 2014, more than 50% of cancer drugs have been approved in multiple indications, and this number is expected to rise to 75% by 2020 [15].

If the benefit of a medicinal product varies according to the indication and the line of treatment, it would be expected that, consequently, its price could also be variable [14, 15]. However, in most healthcare systems today, the price of a drug is the same across indications (except for different dosages) and a single price for all indications may have negative consequences [16]. Ultimately, this practice could lead to the fact that some medicines may never be developed for a given indication, which could constitute a major technological breakthrough, simply because this would lead to a price lower than that set for another indication [14, 15]. The drug may also be used off-label at a much higher price than due. The development of policies that allow the definition of price per indication (PPI) can contribute to a better alignment of the reimbursement with the value of the medicinal product [14, 15] and the application of RSAs by indication may undoubtedly be an alternative as a way of implementing a PPI.

Three modalities to achieve the expected results of PPI are highlighted [15]:

- *Differentiation of medicines according to indication*—For the same medicinal product, the pharmaceutical company establishes separate trade names according to the indication, which are authorised and marketed at different prices;
- *No differentiation with single ‘medium-weighted’ price*—The same medicinal product marketed under a single trade name regardless of the indication, with a single price after weighting the estimated population size for each indication, with possible retrospective reconciliation through discounts based on actual use;
- *No differentiation with specific adjustments by indication*—The same medicinal product, marketed under a single trade name, irrespective of the indication, with separate discounts per indication; one of the possibilities will be to establish different RSAs depending on the indication, resulting in different prices.

Currently, the use and interest in PPI in Europe is still limited [14], unlike the USA where several discussions about this type of price definition are ongoing, to some extent limited by legal and regulatory barriers [14, 16]. Nevertheless, countries such as France and Germany tend to use model b. Others, such as Italy, have been using RSA (model c), especially for cancer drugs, where these agreements allow the use of net prices, that is, prices actually paid, which vary between indications for the same drug under the RSAs. As in Italy, the United Kingdom and Sweden also showed interest in being able to follow this path [14, 16, 17]. Some operational challenges of this model are related to the availability of data and the capacity of managing agreements that involve different net selling prices per indication, requiring monitoring of the volume of use by indication and financial reconciliation *ex post* to ensure that the correct funds flow between the parties, both nationally and sub-nationally [14].

It remains uncertain whether the implementation of PPI agreements will become standard practice, as there is interest from all stakeholders in its potential use, but some scepticism about the NHS ability to obtain good value from its use. At the same time, it will have to be possible to increase the price of a medicinal product if a higher value indication comes after one with a lower value and cost [3].

## Role of RSAs in combinations of medicines

There are more and more drug combinations being tested and approved due to the increasing knowledge about the course of pathologies and their mechanisms of origin [18]. This reality is increasingly relevant in fields such as oncology, human immunodeficiency virus (HIV), hepatitis C and diabetes. When the various drugs can be combined in a single pharmaceutical form, a tablet for example, the exercise on the calculation of the price of the association appears to be relatively simple since the combination is treated as a single product with a different price. However, in most cases, it is not possible to have the combination in a single tablet, or ampoule, and pricing becomes substantially more complex. In addition, in the case of combinations of medicinal products from different companies, the challenges in defining the price and, consequently, in their reimbursement intensify. The fact that the pharmaceutical company owns a single or several components of the combination has implications at stakeholder level and at the variables to be discussed in a negotiation process. The reimbursement procedure for a drug combination will be delineated taking into account multiple factors, such as the number of drugs in the combination considered innovative, the duration of the patent for each of the components or the penetration rate of each drug. It will also be crucial to deal with other, often interdependent and uncertain variables, such as the results of ongoing clinical trials with the combinations and their alternatives, as well as future pricing strategies for competing products [18, 19].

It is also important to assess the impact that the combination will have in terms of duration of treatment compared to the alternatives; and in oncology, this is a central factor, because it is common for a new combination to bring greater survival and consequently a longer period of treatment. RSAs can be of extreme importance in this context, because they allow setting discounts or limits on the costs of the combination, intra- or inter-pharmaceutical company, enabling access to them.

## The importance of registries in the implementation of RSAs

One of the key steps required to leverage the implementation of RSAs is the creation and/or adaptation of information systems which make it possible to collect data on the use of medicines. This collection should take place preferably in an automated manner and integrating existing information systems along the patient’s journey, in order to minimise the additional administrative burden for health professionals and maximise efficiency within institutions. Through these specifics, the system will be able to issue reports in real time, so that monitoring of the use of medicines and the clinical situation of patients can be accessed immediately and continuously.

The first step consists of defining a matrix containing the minimum required parameters to be collected, such as in an observational study or a clinical trial. This selection of variables must inevitably reflect the type of disease and take into account the nature of the various agreements that may be established, as well as the flow of information that potentially will be established between the various entities involved (payer, provider and pharmaceutical company).

Table 2 presents a suggestion of minimum data to be collected in order to implement an RSA for cancer drugs administered intravenously [20]. In this case, the fields displayed are grouped into four categories: patient identifying information, information about the disease, information about the medicinal product, with particular focus on variables that allow for inferring whether it is being used correctly according to the established agreement, and information about the treatment. In this way, it will be possible to monitor the RSA and proceed to the calculation of applicable amounts to be paid back.

It should be noted that the personal data to be collected under these registries should remain only within the entities that create them, and can be shared, in accordance with the law, in an anonymized and/or aggregated manner.

## RSAs in Portugal

Portugal is one of the European countries with specific regulations for reimbursement and funding of medicines, which includes a legal framework for the RSA: Decree Law No. 195/2006 article 5 for medicines for hospital use and Decree-Law No. 48-A/2010 article 6 for retail medicinal products. More recently, the National System of Health Technology Assessment (SiNATS), created in 2015 (Decree Law No. 97/2015), reinforces that health technologies are the object of evaluation and re-evaluation in an integrated context and with preferred resource towards goal setting through contracts with pharmaceutical companies holding the marketing authorisations. The SiNATS provides for the possibility of negotiation and definition of an RSA regarding the use of health technologies.

The literature on the implementation of RSAs in Portugal is still scarce. A survey carried out at the European level [7], in 2013, has identified 84 agreements in Portugal, of which 74 were financial agreements, two were based on clinical outcomes, and eight were mixed. The authors did not find literature that reported the impact of these agreements in Portugal [7]. Since then, in addition to implementing a cap on expenses applicable to all hospital medicines, more recently, there have been announcements of the signing of other types of financial RSAs, including ones based on total medicine cost per patient, as well as others based on clinical results. As such, the following agreements are featured, signed between pharmaceutical companies and the National Authority of Medicine and Health Products (Infarmed) from 2014 until now:

- Vemurafenib (Zelboraf^®^) for the treatment of BRAF V600 mutation-positive unresectable or metastatic melanoma [22];
- Certolizumab pegol (Cimzia^®^), for the treatment of moderate to severe active rheumatoid arthritis, [23];
- Sofosbuvir (Sovaldi^®^) and Ledipasvir + Sofosbuvir (Harvoni^®^) for treatment of chronic hepatitis C [24];
- Pertuzumab (Perjeta^®^) for treatment of HER2-positive metastatic or locally recurrent unresectable breast cancer [25];
- Canacinumab (Ilaris^®^) for treatment of cryopyrin-associated periodic syndromes [26].

Two of these five agreements refer to cancer treatments, which highlights the importance of RSAs in access to these therapies. This list of RSAs is certainly undersized, as it is based on information contained in evaluation reports of hospital drugs reimbursement published by Infarmed, excluding RSAs established in the context of retail drugs or other confidential agreements between companies holding the products directly with national hospitals. In the specific case of RSAs for hepatitis C, and according to a balance sheet of Infarmed, these not only resulted in clinical benefit for patients, but also a reduction in public spending with cost savings in treatment of the consequences of the disease evolution [27–29].

Thus, these agreements represent a valuable strategic tool and allow not only universal access to therapeutic innovation, but also contribution to the sustainability of the NHS [21, 24, 27].

In Portugal, in the framework of RSAs have emerged in several hospitals efforts to develop information systems, and some have managed to implement an integrated and qualified system for the adoption of such agreements. In other hospitals, regardless of implementation of RSA, healthcare professionals including clinicians and in particular, oncologists, are aware of the importance of creating registries and specifically their integration into the electronic clinical process.

## Conclusions

Due to a continuous increase in health expenditure and the rising cost of healthcare, policymakers were compelled to implement various mechanisms to contain this increase and to use resources more efficiently. RSAs can be perceived as part of this comprehensive reaction in view of the sustained increase of healthcare costs and the challenges of access to innovation [1], namely, in Europe and particularly in oncology.

In recent years, the use of RSAs has gained importance in European countries and has denoted a substantial increase of agreements based on results versus purely financial agreements. Regardless of the type of agreement, there are recognised advantages in their adoption, which are inevitably associated with challenges of implementation.

To date there has been no agreement defined as the gold standard [1] and there are still some barriers that need to be overcome, so that the implementation of RSAs is carried out in full [3, 9], such as frequently lengthy negotiation processes, the need for investment, heavy administrative burden, a lack of transparency in the definition of criteria, the need for regulation and skewing of pricing information for international referencing. Another major challenge is the current information systems, the majority of which are inadequate and nonintegrated. It is crucial to draw recommendations and conduct practical assessments on the results of RSAs because, in most cases, it still remains unknown if the objectives initially set out are actually achieved, namely, if RSAs have contributed to an effective control of expenditures, if RSAs resulted in fairer and more timely patient access to therapeutics, if RSAs contributed to improve incentives for innovation, and what the resulting policy implications are [1, 3, 10]. These assessments, well-grounded and carried out systematically, allow us to draw conclusions to improve the design and implementation of future agreements. The absence of a process to evaluate these agreements by the various entities involved can compromise their effectiveness. Nevertheless, RSAs seem to have a relevant role in improving access to innovation, particularly to oncological therapies and with regard to the reduction of the time until authorisation of use or reimbursement [1, 3, 9].

The definition of a price per indication, particularly in oncology, may help health systems towards a better alignment between the reimbursement of medicinal products with their value. However, their adoption in European markets is still variable. One of the ways to implement a price per indication could be by the definition of an RSA by indication. This practice requires specific registries by indication, which doesn’t happen in most countries [15]. Currently, besides Italy [17], there are no countries that routinely set prices that differ by indication, except in the case of multi-brand medicines. In Germany and France, the HTA procedure itself decreases the need to have pricing based on indication. The negotiated price already represents a form of weighted average price for multiple indications [14, 16]. However, in the absence of mechanisms that allow an increase of this average price resulting from the entry of later indications delivering greater clinical benefit, the existing system in these countries may not be suitable, risking under investigation on indications of higher clinical need, simply because the price to be applied would not coincide with the delivery of higher value. Agreements are likely to be developed for indication in the United Kingdom and Sweden [14, 16] and in Portugal it can also be an option to explore.

The emergence of drug combinations, namely, those of innovative drugs from different companies, will be a trend that will inevitably change the paradigm of medicinal products marketing. In oncology, this era of immunotherapies, targeted therapies and/or combinations of both, will pose important challenges with regard to patient access and to the premise of the required health systems sustainability. More sophisticated approaches are needed to formulate strategies for pricing definition and reimbursement that are able to accommodate the impact of a significant number of variables with a high level of uncertainty [18]. There is no short-term solution in sight and therefore pilots at national level for the implementation of pricing strategies for innovative drug combinations in several disease areas, including oncology, may provide learning tools and allow development of a future operation model of access to these combinations in the context of their reimbursement [19].

With regards to the situation in Portugal in terms of RSAs, most are merely financial in nature [3, 7], although it is expected that these will be replaced by agreements based on clinical outcomes. In order for this transition to takes place seamlessly, Portugal must anticipate and drive the creation or urgent updating of computer registries to capture the data needed for the implementation of RSAs based on outcomes. The creation and maintenance of these registries is an obstacle to the expansion of RSAs due to the investment required, either regarding human or financial resources. However, it is indisputable that part of this limitation can be overcome, namely, through the development of a model registry, consisting of several matrixes to be combined and customised according to the specificities of each therapeutic area. This tool would harmonise the data collection process and thereby speed up the process and ease the effort incurred at the time of the creation of a new RSA.

It can be concluded that personalised healthcare will require alternative models that allow for the alignment of price with value, personalised reimbursement models, in order to allow patient access to innovation in a particular indication or combination. There is a need for closer and continued collaboration between the different parties, along with a supporting infrastructure for information sharing.

RSAs should reflect a real commitment to serving the needs of patients, simultaneously allowing for greater choice and ensuring the possibility of access to the most appropriate treatment. This means that the risk may be on the payer’s side, with the pharmaceutical company, or both, but never to the detriment of the patient [1].

The ability to continue to successfully develop RSAs in Portugal, Europe and in the world will certainly be an important step forward in terms of patient access to treatments, particularly but not exclusively in the field of oncology, and a gain in the efficiency of healthcare.

## Conflicts of interest

The authors confirm the thoroughness and accuracy of the contents, and the opinions expressed are the sole responsibility of the authors. Susana Santos works at the company that financed the creation of this article. Catarina Silva works at the company that received payment for carrying out medical writing.

## Figures and Tables

**Table 1.** Advantages and disadvantages of risk-sharing agreements.

Perspective	Advantages	Disadvantages
Patients/society	- Access to innovative medicines - More treatment options and potential health improvement - Promotion of investment for innovation	- Risk of the medicine not displaying the expected benefit - Discontinuation of access to medicine at the end of the agreement - Issues relating to data protection
Providers	- Greater knowledge and improved disease management - Access to innovative medicines - Limiting budgetary impact - Reduction of uncertainty concerning effectiveness	- Costs/bureaucracy of implementation and monitoring of the agreement - Computerisation of data and follow-up of patients complex/costly - Complexity of multiple agreement management
Payers	- Collection of additional evidence (that supports financing decision) - Management of uncertainty (effectiveness and budget) - Therapy directed at patients with potential to benefit (avoiding risk in patients who would not benefit)	- Difficulty in defining easily measurable performance indicators - Lack of integrated information system that allows data collection at local and national level - Intensive allocation of resources in data collection and analysis/monitoring of the agreement
Pharmaceutical companies	- Access of innovative medicines to the market - Improved performance of medicine due to use for target patient - Innovation rewarded and research and development stimulated - Terms of agreement confidential, including price	- Costs/bureaucracy of implementation and monitoring of the agreement - Risk of not demonstrating alleged effectiveness - Financial unpredictability, depending on the type of agreement - Biased selection of patients with worse prognosis

**Table 2.** Minimum data required for implementing risk-sharing agreements.

	Field	Description
Patient information	Anonymised patient ID	Unique patient identification, anonymised
Disease information	Identification of disease	Diagnosis (e.g. early disease, metastatic, unresectable, locally advanced)
	Mutational status	The existence of certain mutations can be important to complete diagnosis and/or opt for certain therapy (e.g. KRAS, RAS, HER2, EGFR, BRAF, ALK)
Medicinal product information	Main medicinal product/co-medicinal product	Identification of the medicinal product administered/medicinal product administered in combination
	Amount administered (main medicinal product/co-medicinal product)	Total dose administered in a given cycle
	Date of administration (main medicinal product/co-medicinal product)	Date on which the administration occurred
	Presentation of the medicinal product administered (main medicinal product/co-medicinal product)	e.g. Medicinal product A 150 mg vial
	Number of units administered (main medicinal product/co-medicinal product)	e.g. 2 vials
Treatment information	Line of treatment	e.g. first line metastatic treatment, second line of metastatic treatment, adjuvant, neoadjuvant treatment
	Treatment status*	Not started, ongoing, finished
	Treatment cycle number	e.g. 1, 2, 3, 4,...
	Date of evaluation of response (intermediate or final evaluation)*	Date of evaluation of response to treatment
	Evaluation of response to treatment (intermediate or final evaluation)*	Description of response to treatment (e.g. complete response, partial response, stable, disease progression, death, not assessable)
	End of treatment date*	Date of the end of treatment with a given medicinal product
	Reason for end of treatment*	Reason for interruption of certain treatment (e.g. treatment completed, progression, death, toxicity, patient decision)

* Information required for agreements based on clinical outcomes
